# Assessment of the Bone Healing Process Mediated by Periosteum-Derived Mesenchymal Stem Cells’ Secretome and a Xenogenic Bioceramic—An In Vivo Study in the Rabbit Critical Size Calvarial Defect Model

**DOI:** 10.3390/ma14133512

**Published:** 2021-06-24

**Authors:** Mindaugas Pranskunas, Egidijus Šimoliūnas, Milda Alksne, Victor Martin, Pedro Sousa Gomes, Algirdas Puisys, Algirdas Kaupinis, Gintaras Juodzbalys

**Affiliations:** 1 Department of Oral and Maxillofacial Surgery, Faculty of Odontology, Medical Academy, Lithuanian University of Health Sciences, LT-50161 Kaunas, Lithuania; gintaras@stilusoptimus.lt; 2 32:Balti Dental Clinic, LT-09235 Vilnius, Lithuania; 3 Life Sciences Center, Department of Biological Models, Institute of Biochemistry, Vilnius University, LT-10257 Vilnius, Lithuania; egidijus.simoliunas@gmail.com (E.Š.); milda.peciukaityte@gmail.com (M.A.); 4 BoneLab—Laboratory for Bone Metabolism and Regeneration, Faculty of Dental Medicine, U. Porto, 4200-393 Porto, Portugal; victorzmartin@gmail.com (V.M.); pgomes@fmd.up.pt (P.S.G.); 5 LAQV/REQUIMTE—U. Porto, 4200-393 Porto, Portugal; 6 Vilnius Implantology Center, LT-03162 Vilnius, Lithuania; algirdas@vicklinika.lt; 7 Vilnius Research Group, LT-02233 Vilnius, Lithuania; 8 Proteomics Centre, Institute of Biochemistry, Life Sciences Center, Vilnius University, Saulėtekio av. 7, LT-10257 Vilnius, Lithuania; algirdas.kaupinis@gf.vu.lt

**Keywords:** secretome, mesenchymal stem cells (MSCs), bone regeneration, bone defect

## Abstract

The mesenchymal stem cell (MSC) secretome has been considered an innovative therapeutic biological approach, able to modulate cellular crosstalk and functionality for enhanced tissue repair and regeneration. This study aims to evaluate the functionality of the secretome isolated from periosteum-derived MSCs, from either basal or osteogenic-induced conditions, in the healing of a critical size calvarial bone defect in the rabbit model. A bioceramic xenograft was used as the vehicle for secretome delivery, and the biological response to the established biocomposite system was assessed by clinical, histological, histomorphometric, and microtomographic analysis. A comparative analysis revealed that the osteogenic-induced secretome presented an increased diversity of proteins, with emphasis on those related to osteogenesis. Microtomographic and histological morphometric analysis revealed that bioceramic xenografts implanted with secretomes enhanced the new bone formation process, with the osteogenic-induced secretome inducing the highest bone tissue formation. The application of the MSC secretome, particularly from osteogenic-induced populations, may be regarded as an effective therapeutic approach to enhance bone tissue healing and regeneration.

## 1. Introduction

Mesenchymal stem cells (MSCs) are a self-renewable and multipotent heterogeneous population, with the ability to adhere to tissue culture plates expressing a spindle-shape morphology [1,2]. These cells have been isolated and expanded from the stroma of distinctive tissues and anatomical locations, showcasing identifying features that reflect their individual and differential origin [3]. Despite their quiescent nature under resting conditions, upon biological stimulation MSCs are activated into a highly proliferative population, believed to be recruited into injured sites to assist on the healing/regeneration of the damaged tissues [4]. MSCs are also easy to isolate and expand in vitro and lack significant immunogenicity, substantiating their relevant application in tissue engineering and regenerative medicine applications [5].

Given the significant reparative/regenerative potential, as well as the improved approaches to minor biosafety concerns and to improve the bioefficacy, MSCs have been considered for bone regenerative strategies [6]. The local injection of MSCs or the implantation of MSCs-loaded scaffolds significantly improved the bone healing process in several experimental and clinical models [7,8]. These therapies facilitated the vascular support through enhanced angiogenesis, diminished inflammation, and promoted a pro-osteogenic microenvironment at the healing site [8]. Despite the enhanced healing outcome, there is limited evidence of the in situ cellular engraftment and the MSC differentiation into the osteogenic lineage functionally contributing to the enhanced bone formation [9]. On the other hand, MSCs seem to induce a significant therapeutic activity via the production of distinct bioactive factors that support and enhance the healing process [10,11].

In this framework, the MSC repertoire of secreted molecules/biological factors—broadly defined as the secretome—has attracted much attention as an innovative therapeutic biological approach able to modulate cellular crosstalk and functionality, enhancing tissue repair and regeneration [10]. Secreted substances include growth factors, cytokines, chemokines, hormones, cell-adhesion molecules, and, in a lower abundance, lipid mediators and nucleic acids, being released through distinctive mechanisms as exocytosis, translocation, and exome or vesicle encapsulation [12,13]. Released factors can act directly on target cells or indirectly on neighboring tissues, showcasing a conjoined enhancement of tissue repair via proangiogenic, anti-inflammatory, anti-apoptotic, and immunomodulatory activities [13,14]. Additionally, secretome-based applications may obviate several biological-related concerns associated with the use of cells (e.g., immune compatibility, disease transmission, and tumorigenicity), as well as sustain distinctive logistic advantages regarding clinical application (e.g., obtainability, scalability, increased shelf life) [15,16].

The secretome’s composition seems to vary regarding species, tissue source and location, physical–chemical stimuli, and microenvironmental characteristics [17]. The secretome from MSCs isolated from the bone marrow and adipose tissue have been further characterized, evidencing effective functionality on the enhancement of the tissue healing, despite the established functional variances and, potentially, differences in the therapeutic outcomes [18,19,20]. A common core of factors released from MSCs from different tissues has been described, broadly embracing constituents of the cell structure and extracellular matrix, and regulators of metabolic functions [21,22], sustaining the importance of the common cellular niche into the improvement of communal biological processes such as cell survival, proliferation, and migration, as well as extracellular matrix organization and immunomodulation. On the other hand, type-specific actions have further been recognized, i.e., visceral adipose tissue MSC secretome has been associated with enhanced detoxification activity, while that from subcutaneous adipose tissue MSCs was further related to an osteo/chondrogenic activity, and the bone marrow-derived MSC secretome was further associated with extracellular matrix remodeling [22,23]. Additionally, differences regarding the potency of the biological modulation induced by different MSC secretomes have also been reported [24].

However, little is known regarding the MSC secretome from alternative sources, such as the periosteum. Periosteum is a connective tissue, organized into a fibrovascular membrane that embraces the bone surface. It consists on an outer layer—rich in elastic and collagenous fibers and developed vascular and neural networks, and an inner cambium layer—highly cellular in nature with progenitor cells, osteoblasts, and fibroblasts, and also rich in vascular and neural networks [25]. Functionally, periosteum contributes to bone elongation and modeling during growth and development, further assisting with bone repair/healing and contributing directly to the bone formation process, as well as indirectly, via paracrine activation [26,27]. In this framework, this study is set on the assessment of an innovative approach for the enhancement of bone healing by (a) addressing the functionality of the secretome isolated from periosteum-derived MSCs as a novel cell source for translational approaches; and (b) characterizing and comparing the functionality of the secretome obtained from either basal or osteogenic-induced cells in the healing of a translational bone defect—the critical size calvarial bone defect in the rabbit model. A commercially biocompatible bioceramic xenograft was used as the vehicle for local secretome delivery, and the biological response to the delivered system was assessed, at distinct time points, by clinical, histological, histomorphometric and microtomographic analysis.

## 2. Materials and Methods

### 2.1. Animals

Eighteen adult male New Zealand White rabbits, weighing between 2750 g and 3000 g, were obtained from a certified dealer and used in this study with the approval of the State Food and Veterinary Service (identification code: G2-55), observing the guidelines for the protection of laboratory animals according to the European guidelines (European Directive 2010/63/EU). Prior to any experimental manipulation, animals were quarantined for 3 weeks. Animals were housed in environmental enriched individual cages. All animal experiments were conducted in compliance with the ARRIVE guidelines. Animals were housed in a temperature-, humidity-, and air renewal-controlled room and accommodated under a 12 h light–dark cycle, according to the guidelines of Annex II of Directive 86/609/EEC. Animals were fed standard dried diet and water ad libitum. Animals were monitored daily throughout the experimental period.

### 2.2. Periosteum MSC Isolation and Characterization

In this study, periosteum-derived MSC were used. As donors, three animals were chosen at random. Donor animals were later included in the experimental groups. The premedication was induced by injection of acepromazine (0.5 mg/kg) in thigh muscles, and a subcutaneous injection of buprenorphine (0.03 mg/kg). General anesthesia was achieved by injection of ketamine hydrochloride (35 mg/kg) and xylazine hydrochloride (5 mg/kg) in thigh muscles. Carbomer eye gel (Oftagel 2,5 mg/g, SANTEN OY, Tempere, Finland) was used to keep the eyes wet. All surgical procedures were performed using a special warming surgical table and special covers to keep the animals warm and achieve better sterility. After shaving the surgical site, it was disinfected with alcohol and local anesthetic with articainum/epinephrinum performed (Ubistesin 40 mg + 5 mcg/mL, 3M Deutschland, Germany). The surgical area was expanded using a sagittal incision through skin and periosteum around the entire thickness, and 5 × 5 mm of periosteum was cut out and transferred to cell growth media (GM) composed of Dulbecco′s Modified Eagle′s Medium (DMEM) (12800-017, Gibco, Thermo Fisher Scientific Waltham, MA, USA), supplemented with 10% fetal bovine serum (FBS) (A31608-01, Gibco, Thermo Fisher Scientific Waltham, MA, USA) and antibiotics: penicillin 100 U/mL and streptomycin 100 mg/mL (5140-122, Gibco, Thermo Fisher Scientific Waltham, MA, USA). Tissue was further processed under sterile laminar flow conditions. Using sterile scissors, tissue was minced into smaller pieces, approximately 1 mm^3^ in size. Periosteum tissue pieces were transferred to the sterile 15 mL vial with 4 mL of 1 mg/mL collagenase A solution prepared in DMEM for 18 h at 37 °C while gently stirring. Then, tissue pieces were separated from the cells by filtrating through a sterile 70 µm nylon mesh sieve. The cell suspension was centrifuged at 400 g for 10 min at 4 °C, the supernatant was then discarded, and cells were suspended in GM. Next, the cell suspension was seeded to 25 or 75 cm^2^ cell culture flasks. Cells were grown in the incubator with 95% humidity and 5% CO_2_ concentration at 37 °C temperature. Cells were divided into new flasks after monolayers reached 70–80% confluence. Cells were dissociated from the tissue culture flask surface with 0.05% trypsin/EDTA solution. Passages 3–6 were used for the experiments.

In order to evaluate periosteum-derived MSCs’ differentiation potential, cells were grown in adipogenic, myogenic, and osteogenic differentiation inducing media (Supplementary Materials, Figure S1). For adipogenic differentiation induction, cells were grown in DMEM supplemented with 10% fetal bovine serum (FBS), 1% penicillin streptomycin solution, 1% glutamine, 1 µM dexamethasone, 1 µM indomethacin, 500 µM 3-isobutyl-1-methylxanthine, and 10 µg/mL human recombinant insulin. Cells were grown in this media for 4 days. Then, cells were fixed with 4% formaldehyde for 10 min at room temperature (RT). Latter fixed cells were washed with distilled water followed by 5 min washing with 60% isopropanol at RT. Lipid reserves stored in the cells were stained with Oil Red O staining solution (0.5% solution in isopropanol) for 10–15 min. After removing the dye, cells were washed with deionized water until the water became clear. Red stained droplets were visualized and captured with a CCD camera (EXi Blue, QImaging, Surrey, British Columbia, Canada) attached to a microscope (IX51, Olympus, Shinjuku, Tokyo, Japan). For myogenic differentiation induction, cells were grown in DMEM supplemented with 2% horse serum and 1% penicillin streptomycin solution, for one week. Cell differentiation was evaluated by visualizing multinuclear cells with crystal violet staining. Briefly, myogenic differentiation media was removed, and cells were washed 3 times with PBS and stained with 0.1% crystal violet solution for 30 min. Then cells were washed with distilled water, and multinuclear cells were visualized and captured with a CCD camera (QImaging EXi Blue) attached to a microscope (Olympus IX51). For osteogenic differentiation, cells were grown as detailed for OsteoSec preparation. Differentiation was carried out for 21 days, and half of the media was replaced every two/three days. Osteogenic differentiation was confirmed by staining formed calcified extracellular matrix with Alizarin Red S (ARS) dye. Osteogenic differentiation media was removed, and cells were fixed with 4% formaldehyde for 10 min at RT. Then cells were washed 3 times with PBS and stained with 2% ARS (pH 4.1–4.2). Cells that formed calcified ECM (stained in red) were visualized and captured with a CCD camera (QImaging EXi Blue) attached to a microscope (Olympus IX51).

### 2.3. Preparation of Secretomes

Two types of secretomes were used in this study—one from undifferentiated MSCs (UndifSec) and another from osteogenic-induced MSCs (OsteoSec). For UndifSec, cells were sown at 75 cm^2^ in Falcon flasks at a density of 40,000 cells/cm^2^. Cells were grown for 21 days in GM. The next day, cell GM was removed, cells were washed three times with PBS, and serum-free DMEM was added. Cells were grown for an additional 3 days, in previously defined conditions. OsteoSec was generated from the same periosteum delivered MSCs. Briefly, cells were seeded as described, but culture media was replaced with osteogenic-inducing media (OM) containing DMEM further supplemented with establish inducers: 5 × 10^−8^ M dexamethasone, 25 μg/mL ascorbic acid, and 10^−2^ M β-glycerophosphate. Cells were grown for 21 days; half of OM was changed 2 times every week. After 21 days, cells were washed three times with PBS, and OM was changed to serum free DMEM, with cells grown for an additional three days. Then, both OsteoSec and UndifSec were collected in 50 mL Falcon tubes and centrifuged for 15 min at 6000 RCF. Supernatant was filtered through 0.22 µm syringe driven PVDF filters to new 50 mL tubes and stored at 4 °C. All secretomes were used up in 30 days after collection.

### 2.4. Sample Preparation for Proteomic Analysis

First, secretome samples were enriched with a ProteoMiner protein enrichment large-capacity kit (Bio-Rad, Hercules, CA, USA), which decreases the amount of high-abundance proteins and enriches medium- and low-abundance proteins. Enrichments were performed according to the manufacturer’s provided protocol. Next, the filter aided sample preparation (FASP) [28] method was used for protein digestion prior to mass spectrometry analyses. Protein lysates were processed by the FASP method using Microcon 30 k centrifugal ultrafiltration units (Millipore) operated at 10,000 g. Briefly, the sample was diluted with 200 μL of 8 M urea (pH 8.5), placed in a filter unit, centrifuged, and washed two times with 100 μL of 8 M urea. Then, 100 μL of 55 mM iodoacetamide was added to the filters, and samples were incubated for 20 min. Filters were washed twice with 100 μL of 8 M urea followed by two washes with 100 μL of 50 mM NH4HCO3 pH 8.0. Protein digestion was then performed by adding trypsin in 50 μL of 50 mM NH4HCO3 at an enzyme to protein ratio of 1:100 and incubating overnight at 37 °C. Peptides were collected from the concentrators by centrifugation at 10,000× *g* for 10 min and additionally eluted using 20% CH3CN. The eluates were combined and acidified with 10% CF3COOH, and peptides were dried in a speed vacuum for 2 h at 45 °C. The lyophilized peptides were redissolved in 0.1% formic acid.

### 2.5. LC–MS-Based Protein Identification

Liquid chromatographic (LC) analysis was performed in a Waters Acquity ultra performance LC system (Waters Corporation, Wilmslow, UK). Peptide separation was performed on an ACQUITY UPLC HSS T3 250 mm analytical column. Data were acquired using a Synapt G2 mass spectrometer (MS) and Masslynx 4.1 software (Waters Corporation, Wilmslow, UK) in positive ion mode using data-independent acquisition (UDMSE). The capillary voltage was set at 2.8 kV, and the source temperature was set at 80 °C. Scan time was set at 0.75 s. Raw data were lock mass-corrected using the doubly charged ion of [Glu1]-fibrinopeptide B (*m/z* 785.8426; [M+2H]^2+^). Raw data files were processed and searched using ProteinLynx Global SERVER (PLGS) version 3.0.1 (Waters Corporation, Wilmslow, UK). Data was analyzed using trypsin as the cleavage protease and one missed cleavage was allowed, and fixed modification was set to carbamidomethylation of cysteines, and variable modification was set to oxidation of methionine. Minimum identification criteria included 1 fragment ion per peptide, and 3 fragment ions and one peptide per protein. The following parameters were used to generate peak lists: (i) low energy threshold was set to 150 counts, (ii) elevated energy threshold was set to 50 counts, (iii) intensity threshold was set to 750 counts. UniprotKB/SwissProt databases were used for protein identification.

### 2.6. In Vivo Evaluation of Secretomes’ Biological Response on the Bone Healing Process

For the evaluation of the biological response to the secretomes in the bone healing process, bioceramic xenograft particles adsorbed with either with UndifSec or OsteoSec were implanted in calvarial bone defects (Table 1). For each defect, 0.5 g of bioceramic particles were used, which were poured into 2 mL of UndifSec, OsteoSec, or DMEM for 5 min. Animals were used for each experimental group, and the bone tissue response was characterized at two time points, 6 and 12 weeks after implantation.

**Figure 1:**
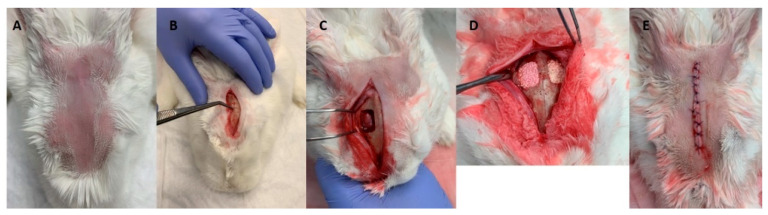
The course of the operation. (**A**) Shaved and disinfected operating area. (**B**) Incision through skin and periosteum. (**C**) Defect formation in 15 × 10 mm size. (**D**) Filling of bone defects. (**E**) Periosteal and skin suturing.

Animals were prepared for the surgical procedure as previously described regarding general management, premedication, anesthesia, and access to the surgical site. Following the elevation of a full-thickness flap at the parietal region, a standardized defect 15 × 10 mm was drilled using a stainless-steel surgical guide and a bone driller (1.5 mm diameter) at low speed and under constant irrigation. Care was taken not to damage the underlying structures (Figure 1). A standardized defect (a total of 36 defects) was established at each parietal bone—2 defects per animal. For each time point, a total of 9 animals was used (*n* = 6 per experimental condition—UndifSec, OsteoSec, and control).

**Figure 2:**
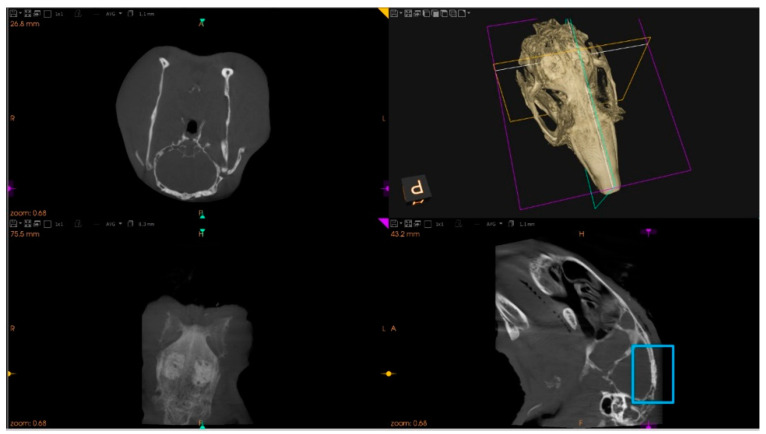
Cone beam computer tomography scan of the skull, done immediately after the surgical procedure.

Established bone defects were filled by the random selection of the condition (i.e., bioceramic xenograft associated with UndifSec, OsteoSec, or DMEM—control). Different test groups were positioned side by side. Then, the incisions were closed in layers with resorbable 5–0 PGA (polyglycolic acid) suture material. Immediately after the surgical procedure, animals were submitted to a cone beam computer tomography scan (CBCT) (Carestream 8100 3D, Carestream Dental LLC, Atlanta, GA, USA) to address the correctness of bioceramic implantation and eventual damage of the cranial structures. Figure 2 indicates the conditions of the CBCT. Postoperatively, pain management was established for 3 days with meloxicam (0.2 mg/kg, IM).

At the end of each defined time point (6 or 12 weeks), animals were euthanized by the administration of a combination of ketamine hydrochloride (50 mg/kg, IM) and xylazine hydrochloride (20 mg/kg, IM), followed by sodium thiopental (25 mg/kg) injection in the marginal ear vein. A systematic necropsy was conducted, and the implanted calvaria region and neighboring tissues were surgically harvested with the surrounding bone and immediately fixed in neutral buffered formalin (10%).

### 2.7. Characterization of the Bone Healing Process

#### 2.7.1. Microtomographic Evaluation

Samples were scanned using a high-resolution micro-CT scan (Skyscan 1172, Bruker microCT NV, Kontig, Belgium). The X-ray source was set at 70 Kv and 141 μA with a voxel size of 13.58 μm and the use of a 0.5 mm Al/0.08 mm Cu filter. Samples were scanned placed in a gauze freshly soaked with 10% formalin and surrounded by a soft plastic sheet. The sample was set on the object stage, and the scan was performed with a 360° rotation, with images acquired every 0.4°. The frame averaging was set at 3 and the random movement at 10. Reconstruction was based on the Feldkamp algorithm using NRecon software (Skyscan 1172, Bruker microCT NV, Kontig, Belgium). For reducing noise smoothing, ring artifacts and beam hardening tools were used and set at 2, 8, and 40%, respectively. Reconstructed images were evaluated with DataViewer software (version 1.5.6.2; Bruker, Bruker microCT NV, Kontig, Belgium) to place the cortical surface of the calvaria perpendicular to the long axis of the ROI to allow the analysis of the bone volume (BV) and graft volume (GV) ratios within the total volume (TV), defined within the margins of the defect, as previously described [29]. Images were loaded in CTAn software (version 1.17.7.2; Bruker microCT NV, Kontig, Belgium) for subsequent evaluation. In order to distinguish between the mineralized phases of the samples—bioceramic xenograft and newly formed bone tissue, a multi-threshold segmentation algorithm was applied, upon histogram partition, in accordance with the comparison between histological images and microtomographic reconstructions, as previously described [30].

#### 2.7.2. Histological and Histomorphometric Evaluation

Following microtomographic evaluation, samples were sectioned and grinded using the Exakt system (Exakt Apparatebau, Hamburg, Germany). Briefly, fixed samples were dehydrated and embedded in an acrylic resin (Technovit 7200 VLC + BPO; Kulzer & Co., Wehrheim, Germany), sectioned, and grinded to sections with a final thickness of about 40 mm. Undecalcified sections were then stained with Levai–Laczko dye for histological and histomorphometric examination with an optical microscope (BX51, Olympus, Tokyo, Japan) connected to a digital color camera (DP71, Olympus, Tokyo, Japan) equipped with a motorized plate (Märzhäuser, Steindorf, Germany). Histomorphometric evaluation was conducted.

### 2.8. Statistical Analysis

Statistical analysis was completed using SPSS software (v. 17, SPSS Inc., Chicago, IL, USA). The new bone ingrowth and graft volume for each healing period are expressed as mean ± standard deviation (SD). The Shapiro–Wilk test was used to analyze the normality of the parameters. Comparison of variables between the 3 groups was performed using a one-way ANOVA model. Pairwise comparisons were performed using the Bonferroni test. All tests were two-sided, and statistical significance was set at *p* < 0.05.

## 3. Results and Discussion

### 3.1. Secretome Characterization

**Figure 3:**
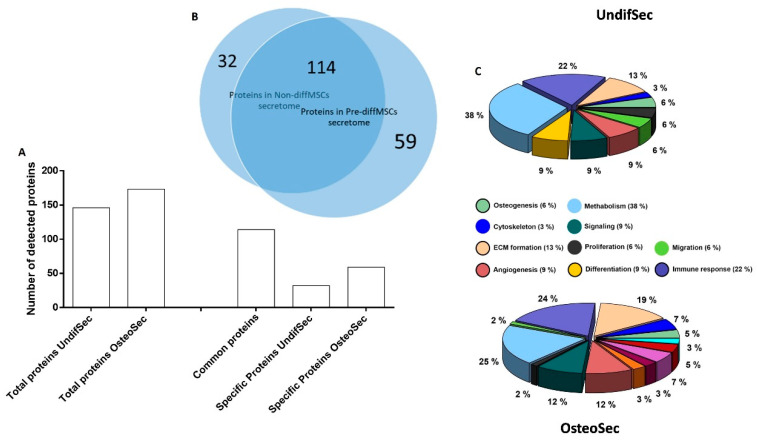
Secretome analysis. (**A**) Frequency of the detected proteins on UndifSec and OsteoSec. (**B**) Venn diagram of the common and specific proteins of UndifSec and OsteoSec. (**C**) Distribution of proteins, according to the biological role, on UndifSec and OsteoSec.

The secretomes of periosteum-derived MSCs, maintained undifferentiated (UndifSec) or osteogenically-induced (OsteoSec), were characterized for protein identification (Figure 3). Regarding UndifSec, a total of 146 distinct proteins were identified, while for OsteoSec, a total of 173 proteins were disclosed. A comparative analysis revealed that 114 were found to be common to both secretomes, while the UndifSec presented 32 unique proteins, and OsteoSec presented 59 unique proteins.

Given the aim of the study, a detailed analysis was focused on the proteins associated with biological function of osteogenesis (Table 1).

The osteogenic induction increased the diversity of secreted proteins (Figure 3), leading to the expression of 27 new proteins, in which collagen alpha-1(I) chain, AE binding protein 1, and stanniocalcin-1 are of particular relevance, given the established relevance in osteogenesis (biological functions of the proteins were verified in the UniProt database). Simultaneously, osteogenic induction suppressed the expression of fibrillin-2 and cathepsin K, within those associated with osteogenesis.

The in vitro induction of the osteogenic program in MSCs, through the supplementation with dexamethasone and ascorbic acid is well established [31] and has been previously found to increase the secretomes’ compositional diversity, particularly in which relates to proteins associated with calcium homeostasis, and extracellular matrix and osteogenic differentiation [32,33]. In the present study, the osteogenic induction led to the selective expression of the collagen alpha-1(I) chain—one component of the fibrillar type I collagen molecule, the major structural constituent the extracellular matrix [34]; AE binding protein 1—a transcriptional repressor of adipogenesis and mitogen-activated protein kinase (MAPK) protector [35], recognized as an effective modulator the osteoblastic differentiation process [36,37]; and stanniocalcin-1, a pleiotropic factor that modulates calcium/phosphate-dependent intracellular signaling and matrix mineralization, further inducing the osteogenic commitment of precursor populations [38,39]. At the same time, the suppression of fibrillin 2—a negative regulator of transforming growth factor beta-(TGF-β) and bone morphogenetic proteins (BMPs)-driven osteoblastic differentiation [40]; and of cathepsin K—a potent cysteine protease that displays a prominent role in mediating bone resorption [41], was attained in the osteogenic-induced secretome. Overall, a synergizing trend to promote the expression of proteins associated with the osteogenic differentiation, and to suppress those associated with osteogenic signaling suppression or bone resorption, were verified, validating the activation of the osteogenic program in differentiating MSCs.

### 3.2. The Bone Healing in Rabbit Calvarial Critical Size Defects

In this study, male New Zealand White rabbits were used, and a critical size calvarial bone defect was selected as the experimental surgical model.

Rabbits offer a high bone turnover rate, a skeletal accretion profile, peak bone mass, and a bone composition comparable to those of humans, and the ability to achieve true skeletal maturity [42,43]. Anatomical features of the calvarial region allow the establishment of suitable and reproducible defects for the characterization of graft materials and the assessment of their influence on the bone healing process [44]. Furthermore, critical size defects (CSDs)—those with the smallest size that do not show spontaneously healing when left untreated for a defined period of time [45], sustain a prototype of discontinuity defects, as a condition of unsuccessful healing to overcome the threshold of the biological process of healing/regeneration [46].

In the present study, two full-thickness defects were created on the calvarial region. The surgical establishment of multiple defects per animal is intrinsically engrained in the 3Rs policy in animal research—Reduction, Refinement, and Replacement—allowing the use of less experimental animals, further minimizing the risk of observational errors, and found not to alter the bone-healing rate, as comparing to the assessment of a single calvarial defect per animal [47]. In addition, the lateral location of the defects, precluding the involvement of the midsagittal suture, further withdraws its possible influence on the healing process and minimizes the risk of sagittal sinus damage [48]. Regarding size, the 15 × 10 mm rectangular defects are recognized CSDs up to 12 weeks of healing [47,49], the second time point selected for characterization of the present study, thus sustaining the present analysis in validated CSDs.

Animals submitted to the surgical calvarial implantation of the bioceramic xenografts, associated or not with UndifSec or OsteoSec, revealed no post-operative complications throughout the experimental period, presenting an adequate recovery. All animals were monitored for behavior and physiological functions (i.e., gastrointestinal and urinary activity), being further submitted to a clinical examination prior to euthanasia. Upon euthanasia, macroscopic observation of the surgical area revealed no altered tissue structure (i.e., infection, ulceration, or abnormal tissue organization), and the conducted systematic necropsy revealed no significant morphological or topographic alterations of the internal organs, i.e., inflammation, degeneration, fibrosis, or necrosis.

### 3.3. Secretome Modulation of the Bone Healing Process

In this study, Cerabone^®^ (Botiss Biomaterials GmbH, Zossen, Germany) was used as a vehicle for the secretomes. Cerabone^®^ is a particulate bioceramic xenograft, prepared from bovine femoral heads, submitted to complex physical–chemical purification methods, including the thermal treatment with temperatures up to 1250 °C [50,51]. It has a validated biocompatibility and has been applied with success in distinct settings, sustaining an enhancement of the bone healing and regeneration processes, in both experimental and clinical applications [52,53,54].

**Figure 4:**
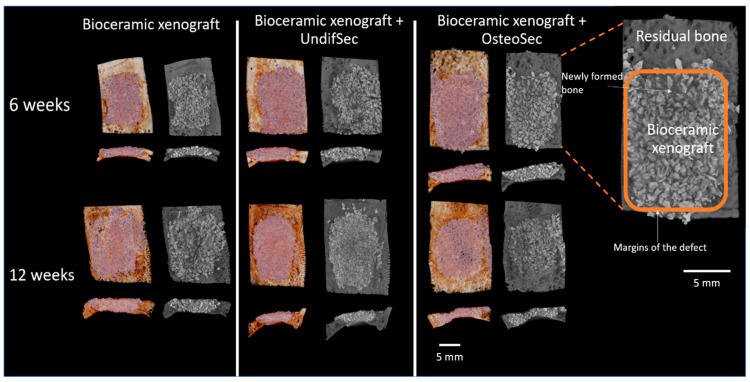
Representative microtomographic reconstructions of the implanted bioceramic xenograft in the absence (control) or presence of the MSC undifferentiated (UndifSec) and osteogenic-induced (OsteoSec) secretomes, for 6 and 12 weeks (*n* = 6).

Implanted calvarial bone defects were characterized by microtomography, which allowed the detailed evaluation of the new bone formation process, as well as the interaction of the bone tissue with the bioceramic, associated or not with the secretomes. The 3D reconstructed images (Figure 4) revealed the proper adaptation of the particulate graft within the established defect, with a progressive integration within the newly formed bone matrix, throughout the 12 weeks evaluation period, within all experimental conditions and controls. Cross-sectional images reveal the complete fill of the defect, with bioceramic xenograft interspersed with newly formed bone. The representative magnified image highlights the graft integration with the scattered mineralized tissue throughout the defect volume.

**Figure 5:**
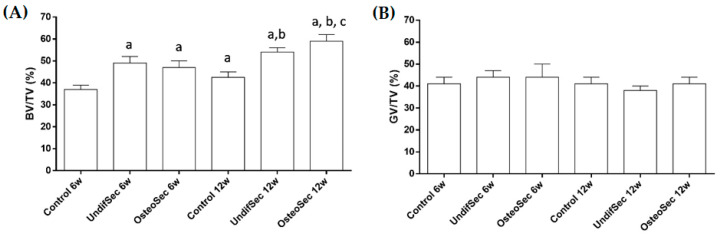
(**A**) Histomorphometric analysis of microtomographic data regarding the newly formed bone tissue (BV/TV) and (**B**) residual bioceramic graft volume (GV/TV) within implanted bioceramic xenograft in the absence (control) and presence of the MSC undifferentiated (UndifSec) and osteogenic-induced (OsteoSec) secretomes, for 6 and 12 weeks. a—significantly different from control 6 weeks, b—significantly different from control 12 weeks, c—significantly different from UndifSec 12 weeks (*n* = 6).

Histomorphometric analysis (Figure 5) focused on the evaluation of the newly formed bone volume within the total tissue volume (BV/TV) and residual bioceramic graft volume within the total tissue volume (GV/TV). The BV/TV was found to increase in all experimental conditions from the 6 to the 12 weeks’ time points. A comparative analysis revealed, for the 6 weeks’ time point, a significant higher bone volume for the conditions loaded with UndifSec or OsteoSec, as comparatively to control. No significant differences were identified between UndifSec and Osteosec. At the 12 weeks’ time point, the conditions implanted with both secretomes presented an increased newly formed bone volume, as compared to control, with the OsteoSec condition presenting the significantly highest value. Regarding GV/TV, no significant differences were attained between experimental conditions or time points, suggesting minimal resorption of the implanted bioceramic xenograft, until the 12 weeks’ time point.

**Figure 6:**
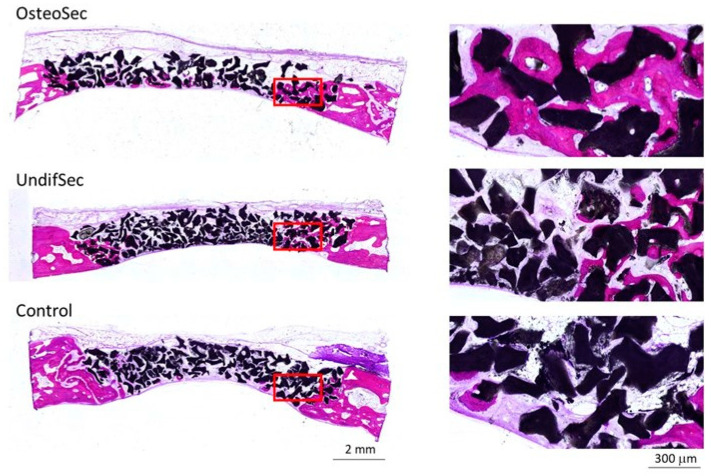
Representative histological sections of the implanted bioceramic xenograft in the absence (control) or presence of the MSC undifferentiated (UndifSec) and osteogenic-induced (OsteoSec) secretomes, for 6 weeks. High magnification images correspond to the inset areas of the low magnification images (*n* = 6).

**Figure 7:**
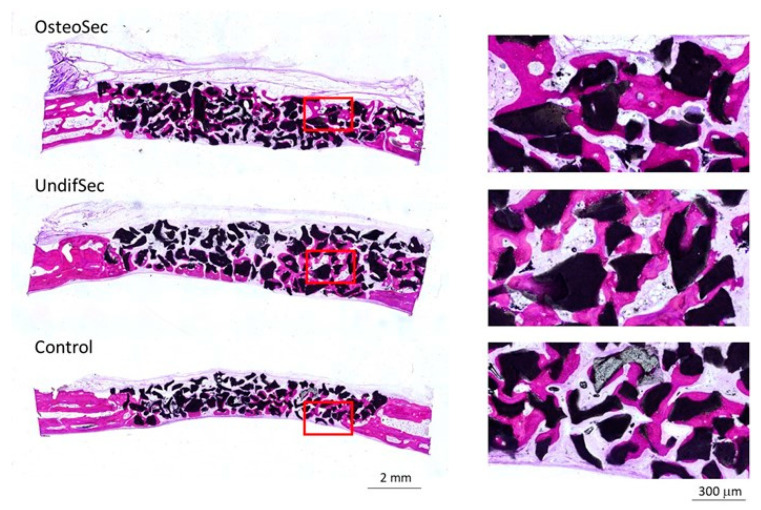
Representative histological sections of the implanted bioceramic xenograft in the absence (control) or presence of the MSC undifferentiated (UndifSec) and osteogenic-induced (OsteoSec) secretomes, for 12 weeks. High magnification images correspond to the inset areas of the low magnification images.

Implanted bioceramic xenografts were further characterized by undecalcified histological techniques. Representative micrographs of all the experimental groups, after 6 and 12 weeks of healing, are shown in Figure 6 and Figure 7, respectively.

**Figure 8:**
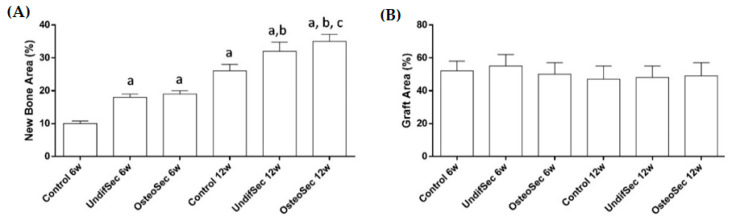
(**A**) Histomorphometric analysis of histological data regarding the New Bone Area and (**B**) Graft Area, for the implanted bioceramic xenograft in the absence (control) or presence of the MSC undifferentiated (UndifSec) and osteogenic-induced (OsteoSec) secretomes, for 6 and 12 weeks. a—significantly different from control 6 weeks, b—significantly different from control 12 weeks, c—significantly different from UndifSec 12 weeks (*n* = 6).

Histological assessment revealed no adverse biological responses (i.e., foreign body reaction, necrosis, or degeneration). At 6 weeks, a discrete new bone formation process was identified, particularly at the margins of the defect, at the control group. Bioceramic implantation with secretomes induced the biological response, with higher levels of new bone formation, particularly evident within the OsteoSec condition (Figure 8). Histomorphometric data sustains the increased bone formation process with the conditions loaded with the secretomes, reporting a significantly increased bone formation, as compared to the control. At the 12 weeks’ time point (Figure 8), an increased newly formed mineralized bone tissue was identified at the defect margin, growing centripetally into the center of the defect, within all experimental conditions. Comparatively, bioceramic xenografts implanted with secretomes appear to induce an increased bone formation process, which is in line with the attained histomorphometric analysis (Figure 7), further showcasing the highest formation of mineralized bone tissue in the OsteoSec condition. Comparatively, no significant differences were attained regarding the bone graft area between conditions or time points.

Overall, bioceramic xenografts implanted with secretomes were found to enhance the new bone formation process, with Osteosec inducing the highest bone tissue formation.

MSCs have been characterized for their secretory ability of distinct bioactive molecules (e.g., cytokines, chemokines, growth factors, proteins, and extracellular vesicles) into the surrounding media—the secretome—with an established modulatory role in a wide range of biological processes such as angiogenesis, extracellular matrix synthesis, and remodeling, further contributing to cell homing and differentiation [55]. Different studies support that the MSC secretomes, administered locally, significantly enhance the bone healing/regeneration process in distinct experimental in vivo settings [56,57,58,59,60,61]. Briefly, the secretome of bone marrow-derived mesenchymal stem cells was found to enhance the bone healing of rat critical size calvarial defects [58,59], while that from amnion-derived multipotent progenitor cells was found to enhance the angiogenesis and the new bone volume formation on rat critical size calvarial defects [56]. Mechanistically, secretome implantation is expected to mobilize and recruit endogenous precursor populations that promote angiogenesis and enhance bone healing outcomes [62,63]. This is line with the data attained in the present study, in which the addition of either secretomes significantly enhanced the bone formation process. The MSC secretome was found to contain factors correlating with angiogenesis and osteogenesis, thus plausibly contributing to the enhancement of bone healing [64]. Notwithstanding, few data are available on the in vivo biological effect of the MSC secretome from osteogenic-induced populations. We verified the increased expression of osteogenic-inducing biomolecules (i.e., collagen alpha-1(I) chain, AE binding protein 1, and stanniocalcin-1), and the suppression of negative regulators or molecules associated with bone resorption (i.e., fibrillin-2 and cathepsin K), which is in line with previous in vitro data [33,65]. These findings seem to support the verified enhancement of the in vivo bone formation process attained with OsteoSec. Taken together, our study provides a comparative analysis of the biological role of the MSC secretome—from either undifferentiated and osteogenic-differentiated conditions—within the bone healing process, showcasing the increased enhancement of the bone regenerative process with the use of the osteogenic-induced MSC secretome.

## Figures and Tables

**Table 1:** Distribution of osteogenesis-promoting proteins in groups.

Osteogenesis-Related Proteins Identified on the Periosteum-Derived MSCs Secretomes		
Common to UndifSec and OsteoSec	Specific to UndifSec	Specific to OsteoSec
Actinin alpha 4 Annexin A2 Collagen alpha-2(I) chain Peroxiredoxin-1 Collagen alpha-1(XII) chain Chitinase-3-like protein 1 Fibrillin-1 Macrophage colony-stimulating factor 1 receptor Pro-low-density lipoprotein receptor-related protein 1	Fibrillin-2 Cathepsin K	Collagen alpha-1(I) chain AE binding protein 1 Stanniocalcin-1

## Data Availability

The data presented in this study are available on request from the corresponding author after obtaining permission of authorized person.

## Supplementary Materials

The following are available online at https://www.mdpi.com/article/10.3390/ma14133512/s1, Figure S1: Evaluation MSC differentiation potential. Cells were differentiated to adipogenic, myogenic and osteogenic lineage. Control column—cells stained with the same dyes but were grown in growth media without differentiation inducing supplements, differentiated column—cells were grown in differentiation inducing media. White circle marks cells with fused/multiple nuclei after myogenic differentiation.

## References

[1] Fierabracci A., del Fattore A., Luciano R., Muraca M., Teti A., Muraca M. (2015). Recent Advances in Mesenchymal Stem Cell Immunomodulation: The Role of Microvesicles. Cell Transplant..

[2] Dominici M., le Blanc K., Mueller I., Slaper-Cortenbach I., Marini F.C., Krause D.S., Deans R.J., Keating A., Prockop D.J., Horwitz E.M. (2006). Minimal Criteria for Defining Multipotent Mesenchymal Stromal Cells. The International Society for Cellular Therapy Position Statement. Cytotherapy.

[3] Sacchetti B., Funari A., Remoli C., Giannicola G., Kogler G., Liedtke S., Cossu G., Serafini M., Sampaolesi M., Tagliafico E. (2016). No Identical “Mesenchymal Stem Cells” at Different Times and Sites: Human Committed Progenitors of Distinct Origin and Differentiation Potential Are Incorporated as Adventitial Cells in Microvessels. Stem Cell Rep..

[4] Wu Y., Chen L., Scott P.G., Tredget E.E. (2007). Mesenchymal Stem Cells Enhance Wound Healing Through Differentiation and Angiogenesis. Stem Cells.

[5] Pacini S. (2014). Deterministic and Stochastic Approaches in the Clinical Application of Mesenchymal Stromal Cells (MSCs). Front. Cell Dev. Biol..

[6] Yong K.W., Choi J.R., Dolbashid A.S., Wan Safwani W.K.Z. (2018). Biosafety and Bioefficacy Assessment of Human Mesenchymal Stem Cells: What Do We Know so Far?. Regen. Med..

[7] Ismail H.D., Phedy P., Kholinne E., Djaja Y.P., Kusnadi Y., Merlina M., Yulisa N.D. (2016). Mesenchymal Stem Cell Implantation in Atrophic Nonunion of the Long Bones: A Translational Study. Bone Jt. Res..

[8] Lin H., Sohn J., Shen H., Langhans M.T., Tuan R.S. (2019). Bone Marrow Mesenchymal Stem Cells: Aging and Tissue Engineering Applications to Enhance Bone Healing. Biomaterials.

[9] Kotobuki N., Katsube Y., Katou Y., Tadokoro M., Hirose M., Ohgushi H. (2008). In Vivo Survival and Osteogenic Differentiation of Allogeneic Rat Bone Marrow Mesenchymal Stem Cells (MSCs). Cell Transplant..

[10] Madrigal M., Rao K.S., Riordan N.H. (2014). A Review of Therapeutic Effects of Mesenchymal Stem Cell Secretions and Induction of Secretory Modification by Different Culture Methods. J. Transl. Med..

[11] Wee Yeh Yeo R., Chai Lai R., Hian Tan K., Kiang Lim S. (2013). Exosome: A Novel and Safer Therapeutic Refinement of Mesenchymal Stem Cell. J. Circ. Biomark. Exosome.

[12] Zullo J., Matsumoto K., Xavier S., Ratliff B., Goligorsky M.S. (2015). The Cell Secretome, a Mediator of Cell-to-Cell Communication. Prostaglandins Other Lipid Mediat..

[13] Tran C., Damaser M.S. (2015). Stem Cells as Drug Delivery Methods: Application of Stem Cell Secretome for Regeneration. Adv. Drug Deliv. Rev..

[14] Kusuma G.D., Carthew J., Lim R., Frith J.E. (2017). Effect of the Microenvironment on Mesenchymal Stem Cell Paracrine Signaling: Opportunities to Engineer the Therapeutic Effect. Stem Cells Dev..

[15] Lukomska B., Stanaszek L., Zuba-Surma E., Legosz P., Sarzynska S., Drela K. (2019). Challenges and Controversies in Human Mesenchymal Stem Cell Therapy. Stem Cells Int..

[16] Vizoso F.J., Eiro N., Cid S., Schneider J., Perez-Fernandez R. (2017). Mesenchymal Stem Cell Secretome: Toward Cell-Free Therapeutic Strategies in Regenerative Medicine. Int. J. Mol. Sci..

[17] Daneshmandi L., Shah S., Jafari T., Bhattacharjee M., Momah D., Saveh-Shemshaki N., Lo K.W.H., Laurencin C.T. (2020). Emergence of the Stem Cell Secretome in Regenerative Engineering. Trends Biotechnol..

[18] Hsiao S.T.F., Asgari A., Lokmic Z., Sinclair R., Dusting G.J., Lim S.Y., Dilley R.J. (2012). Comparative Analysis of Paracrine Factor Expression in Human Adult Mesenchymal Stem Cells Derived from Bone Marrow, Adipose, and Dermal Tissue. Stem Cells Dev..

[19] Petrenko Y., Vackova I., Kekulova K., Chudickova M., Koci Z., Turnovcova K., Kupcova Skalnikova H., Vodicka P., Kubinova S. (2020). A Comparative Analysis of Multipotent Mesenchymal Stromal Cells Derived from Different Sources, with a Focus on Neuroregenerative Potential. Sci. Rep..

[20] Pires A.O., Mendes-Pinheiro B., Teixeira F.G., Anjo S.I., Ribeiro-Samy S., Gomes E.D., Serra S.C., Silva N.A., Manadas B., Sousa N. (2016). Unveiling the differences of secretome of human bone marrow mesenchymal stem cells, adipose tissue-derived stem cells, and human umbilical cord perivascular cells: A proteomic analysis. Stem Cells Dev..

[21] Shin S., Lee J., Kwon Y., Park K.S., Jeong J.H., Choi S.J., Bang S.I., Chang J.W., Lee C. (2021). Comparative Proteomic Analysis of the Mesenchymal Stem Cells Secretome from Adipose, Bone Marrow, Placenta and Wharton’s Jelly. Int. J. Mol. Sci..

[22] Ayaz-Guner S., Alessio N., Acar M.B., Aprile D., Özcan S., di Bernardo G., Peluso G., Galderisi U. (2020). A Comparative Study on Normal and Obese Mice Indicates That the Secretome of Mesenchymal Stromal Cells Is Influenced by Tissue Environment and Physiopathological Conditions. Cell Commun. Signal..

[23] Villatoro A.J., Alcoholado C., Martín-Astorga M.C., Fernández V., Cifuentes M., Becerra J. (2019). Comparative Analysis and Characterization of Soluble Factors and Exosomes from Cultured Adipose Tissue and Bone Marrow Mesenchymal Stem Cells in Canine Species. Vet. Immunol. Immunopathol..

[24] Konala V.B.R., Bhonde R., Pal R. (2020). Secretome Studies of Mesenchymal Stromal Cells (MSCs) Isolated from Three Tissue Sources Reveal Subtle Differences in Potency. In Vitro Cell. Dev. Biol. Anim..

[25] Dwek J.R. (2010). The Periosteum: What Is It, Where Is It, and What Mimics It in Its Absence?. Skelet. Radiol..

[26] Hutmacher D.W., Sittinger M. (2003). Periosteal Cells in Bone Tissue Engineering. Tissue Eng..

[27] Ferretti C., Borsari V., Falconi M., Gigante A., Lazzarini R., Fini M., di Primio R., Mattioli-Belmonte M. (2012). Human Periosteum-Derived Stem Cells for Tissue Engineering Applications: The Role of VEGF. Stem Cell Rev. Rep..

[28] Wiśniewski J.R., Zougman A., Nagaraj N., Mann M. (2009). Universal Sample Preparation Method for Proteome Analysis. Nat. Methods.

[29] Silva T., Silva J.C., Colaco B., Gama A., Duarte-Araújo M., Fernandes M.H., Bettencourt A., Gomes P. (2018). In Vivo Tissue Response and Antibacterial Efficacy of Minocycline Delivery System Based on Polymethylmethacrylate Bone Cement. J. Biomater. Appl..

[30] Daugela P., Pranskunas M., Juodzbalys G., Liesiene J., Baniukaitiene O., Afonso A., Sousa Gomes P. (2018). Novel Cellulose/Hydroxyapatite Scaffolds for Bone Tissue Regeneration: In Vitro and in Vivo Study. J. Tissue Eng. Regen. Med..

[31] Coelho M.J., Fernandes M.H. (2000). Human Bone Cell Cultures in Biocompatibility Testing. Part II: Effect of Ascorbic Acid, β-Glycerophosphate and Dexamethasone on Osteoblastic Differentiation. Biomaterials.

[32] Choi Y.A., Lim J., Kim K.M., Acharya B., Cho J.Y., Bae Y.C., Shin H.I., Kim S.Y., Park E.K. (2010). Secretome Analysis of Human BMSCs and Identification of SMOC1 as an Important ECM Protein in Osteoblast Differentiation. J. Proteome Res..

[33] Kim J.M., Kim J., Kim Y.H., Kim K.T., Ryu S.H., Lee T.G., Suh P.G. (2013). Comparative Secretome Analysis of Human Bone Marrow-Derived Mesenchymal Stem Cells during Osteogenesis. J. Cell. Physiol..

[34] Blair H.C., Larrouture Q.C., Li Y., Lin H., Beer-Stoltz D., Liu L., Tuan R.S., Robinson L.J., Schlesinger P.H., Nelson D.J. (2017). Osteoblast Differentiation and Bone Matrix Formation in Vivo and in Vitro. Tissue Eng. Part B Rev..

[35] Kim S.W., Muise A.M., Lyons P.J., Ro H.S. (2001). Regulation of Adipogenesis by a Transcriptional Repressor That Modulates MAPK Activation. J. Biol. Chem..

[36] Rodríguez-Carballo E., Gámez B., Ventura F. (2016). P38 MAPK Signaling in Osteoblast Differentiation. Front. Cell Dev. Biol..

[37] Ohno I., Hashimoto J., Shimizu K., Takaoka K., Ochi T., Matsubara K., Okubo K. (1996). A CDNA Cloning of Human AEBP1 from Primary Cultured Osteoblasts and Its Expression in a Differentiating Osteoblastic Cell Line. Biochem. Biophys. Res. Commun..

[38] Yoshiko Y., Aubin J.E. (2004). Stanniocalcin 1 as a Pleiotropic Factor in Mammals. Peptides.

[39] Yoshiko Y., Maeda N., Aubin J.E. (2003). Stanniocalcin 1 Stimulates Osteoblast Differentiation in Rat Calvaria Cell Cultures. Endocrinology.

[40] Smaldone S., Ramirez F. (2016). Fibrillin Microfibrils in Bone Physiology. Matrix Biol..

[41] Dai R., Wu Z., Chu H.Y., Lu J., Lyu A., Liu J., Zhang G. (2020). Cathepsin K: The Action in and Beyond Bone. Front. Cell Dev. Biol..

[42] Norris S.A., Pettifor J.M., Gray D.A., Buffenstein R. (2001). Calcium Metabolism and Bone Mass in Female Rabbits during Skeletal Maturation: Effects of Dietary Calcium Intake. Bone.

[43] Djasim U.M., Wolvius E.B., van Neck J.W., Weinans H., van der Wal K.G.H. (2007). Recommendations for Optimal Distraction Protocols for Various Animal Models on the Basis of a Systematic Review of the Literature. Int. J. Oral Maxillofac. Surg..

[44] Frame J.W. (1980). A Convenient Animal Model for Testing Bone Substitute Materials. J. Oral Surg..

[45] Cooper G.M., Mooney M.P., Gosain A.K., Campbell P.G., Losee J.E., Huard J. (2008). Testing the Critical Size in Calvarial Bone Defects: Revisiting the Concept of a Critical-Size Defect. Plast. Reconstr. Surg..

[46] Gomes P.S., Fernandes M.H. (2011). Rodent Models in Bone-Related Research: The Relevance of Calvarial Defects in the Assessment of Bone Regeneration Strategies. Lab. Anim..

[47] Delgado-Ruiz R.A., Calvo-Guirado J.L., Romanos G.E. (2015). Critical Size Defects for Bone Regeneration Experiments in Rabbit Calvariae: Systematic Review and Quality Evaluation Using ARRIVE Guidelines. Clin. Oral Implant. Res..

[48] Bosch C., Melsen B., Gibbons R., Vargervik K. (1996). Human Recombinant Transforming Growth Factor-Beta 1 in Healing of Calvarial Bone Defects. J. Craniofacial Surg..

[49] Haddad A.J., Peel S.A.F., Clokie C.M.L., Sándor G.K.B. (2006). Closure of Rabbit Calvarial Critical-Sized Defects Using Protective Composite Allogeneic and Alloplastic Bone Substitutes. J. Craniofacial Surg..

[50] Seidel P., Dingeldein E. (2004). Cerabone^®^—Eine Spongiosa-Keramik Bovinen Ursprungs. Mater. Werkst..

[51] Murugan R., Rao K.P., Sampath Kumar T.S. (2003). Heat-Deproteinated Xenogeneic Bone from Slaughterhouse Waste: Physico-Chemical Properties. Bull. Mater. Sci..

[52] Mahesh L., Mascarenhas G., Bhasin M.T., Guirado C., Juneja S. (2020). Histological Evaluation of Two Different Anorganic Bovine Bone Matrixes in Lateral Wall Sinus Elevation Procedure: A Retrospective Study. Natl. J. Maxillofac. Surg..

[53] Catros S., Sandgren R., Pippenger B., Fricain J., Herber V., el Chaar E. (2020). A Novel Xenograft Bon e Substitute Supports Stable Bone Formation in Circumferential Defects Around Dental Implants in Minipigs. Int. J. Oral Maxillofac. Implant..

[54] Kapogianni E., Barbeck M., Jung O., Arslan A., Kuhnel L., Xiong X., Krastev R., Friedrich R., Schnettler R., Fienitz T. (2019). Comparison of Material-Mediated Bone Regeneration Capacities of Sintered and Non-Sintered Xenogeneic Bone Substitutes via 2D and 3D Data. In Vivo.

[55] Cooper L.F., Ravindran S., Huang C.C., Kang M. (2020). A Role for Exosomes in Craniofacial Tissue Engineering and Regeneration. Front. Physiol..

[56] Burdette A.J., Guda T., Thompson M.E., Banas R., Sheppard F. (2018). A Novel Secretome Biotherapeutic Influences Regeneration in Critical Size Bone Defects. J. Craniofacial Surg..

[57] Fomby P., Cherlin A.J., Hadjizadeh A., Doillon C.J., Sueblinvong V., Weiss D.J., Bates J.H.T., Gilbert T., Liles W.C., Lutzko C. (2010). Stem Cells and Cell Therapies in Lung Biology and Diseases: Conference Report. Ann. Am. Thorac. Soc..

[58] Chang W., Kim R., Park S.I., Jung Y.J., Ham O., Lee J., Kim J.H., Oh S., Lee M.Y., Kim J. (2015). Enhanced Healing of Rat Calvarial Bone Defects with Hypoxic Conditioned Medium from Mesenchymal Stem Cells through Increased Endogenous Stem Cell Migration via Regulation of ICAM-1 Targeted-MicroRNA-221. Mol. Cells.

[59] Katagiri W., Osugi M., Kawai T., Ueda M. (2013). Novel Cell-Free Regeneration of Bone Using Stem Cell–Derived Growth Factors. Int. J. Oral Maxillofac. Implant..

[60] Ando Y., Matsubara K., Ishikawa J., Fujio M., Shohara R., Hibi H., Ueda M., Yamamoto A. (2014). Stem Cell-Conditioned Medium Accelerates Distraction Osteogenesis through Multiple Regenerative Mechanisms. Bone.

[61] Tsuchiya S., Hara K., Ikeno M., Okamoto Y., Hibi H., Ueda M. (2013). Rat Bone Marrow Stromal Cell–Conditioned Medium Promotes Early Osseointegration of Titanium Implants. Int. J. Oral Maxillofac. Implant..

[62] Osugi M., Katagiri W., Yoshimi R., Inukai T., Hibi H., Ueda M. (2012). Conditioned Media from Mesenchymal Stem Cells Enhanced Bone Regeneration in Rat Calvarial Bone Defects. Tissue Eng. Part A.

[63] Ogata K., Osugi M., Kawai T., Wakayama Y., Sakaguchi K., Nakamura S., Katagiri W. (2018). Secretomes of Mesenchymal Stem Cells Induce Early Bone Regeneration by Accelerating Migration of Stem Cells. J. Oral Maxillofac. Surgery, Med. Pathol..

[64] Gugliandolo A., Fonticoli L., Trubiani O., Rajan T.S., Marconi G.D., Bramanti P., Mazzon E., Pizzicannella J., Diomede F. (2021). Oral Bone Tissue Regeneration: Mesenchymal Stem Cells, Secretome, and Biomaterials. Int. J. Mol. Sci..

[65] Kristensen L.P., Chen L., Overbeck Nielsen M., Qanie D.W., Kratchmarova I., Kassem M., Andersen J.S. (2012). Temporal Profiling and Pulsed SILAC Labeling Identify Novel Secreted Proteins During. Mol. Cell. Proteom..

