# Supplementary material for: Assessment of the Bone Healing Process Mediated by Periosteum-Derived Mesenchymal Stem Cells’ Secretome and a Xenogenic Bioceramic—An In Vivo Study in the Rabbit Critical Size Calvarial Defect Model
Source: Materials (Basel). 2021 Jun 24;14(13):3512. doi: 10.3390/ma14133512 (PMC8269548; doi:10.3390/ma14133512)
Supplement: Supplementary file 1 [file materials-14-03512-s001.zip › materials-1269689-supplementary.pdf]

materials

MDPI

Article

Assessment of the Bone Healing Process Mediated by Periosteum-Derived Mesenchymal Stem Cells' Secretome and a Xenogenic Bioceramic—An in Vivo Study in the Rabbit Critical Size Calvarial Defect Model

Mindaugas Pranskunas [1,2,*,†], Egidijus Šimoliūnas [3,†], Milda Alksne [3,†], Victor Martin [4,5,†], Pedro Sousa Gomes [4,5,†], Algirdas Puisys [6,7], Algirdas Kaupinis [8] and Gintaras Juodzbalys [1,†]

[1] Department of Oral and Maxillofacial Surgery, Faculty of Odontology, Medical Academy, Lithuanian University of Health Sciences, LT-50161 Kaunas, Lithuania; gintaras@stilusoptimus.lt
[2] 32:Balti Dental Clinic, LT-09235 Vilnius, Lithuania
[3] Life Sciences Center, Department of Biological Models, Institute of Biochemistry, Vilnius University, LT-10257 Vilnius, Lithuania; egidijus.simoliunas@gmail.com (E.Š.); milda.peciukaityte@gmail.com (M.A.)
[4] BoneLab—Laboratory for Bone Metabolism and Regeneration, Faculty of Dental Medicine, U. Porto, 4200-393 Porto, Portugal; victorzmartin@gmail.com (V.M.); pgomes@fmd.up.pt (P.S.G.)
[5] LAQV/REQUIMTE—U. Porto, 4200-393 Porto, Portugal
[6] Vilnius Implantology Center, LT-03162 Vilnius, Lithuania; algirdas@vicklinika.lt
[7] Vilnius Research Group, LT-02233 Vilnius, Lithuania
[8] Proteomics Centre, Institute of Biochemistry, Life Sciences Center, Vilnius University, Saulėtekio av. 7, LT-10257 Vilnius, Lithuania; algirdas.kaupinis@gf.vu.lt
* Correspondence: mindaugaspranskunas@hotmail.lt
† These authors contributed equally to this work.

Citation: Pranskunas, M.; Šimoliūnas, E.; Alksne, M.; Martin, V.; Gomes, P.S.; Puisys, A.; Kaupinis, A.; Juodzbalys, G. Assessment of the Bone Healing Process Mediated by Periosteum-Derived Mesenchymal Stem Cells' Secretome and a Xenogenic Bioceramic—An in Vivo Study in the Rabbit Critical Size Calvarial Defect Model. *Materials* **2021**, *14*, 3512. https://doi.org/10.3390/3512

Academic Editor(s): Antonio Scarano

Received: 5 June 2021
Accepted: 22 June 2021
Published: 24 June 2021

Publisher's Note: MDPI stays neutral with regard to jurisdictional claims in published maps and institutional affiliations.

Copyright: © 2021 by the authors. Licensee MDPI, Basel, Switzerland. This article is an open access article distributed under the terms and conditions of the Creative Commons Attribution (CC BY) license (http://creativecommons.org/licenses/by/4.0/).

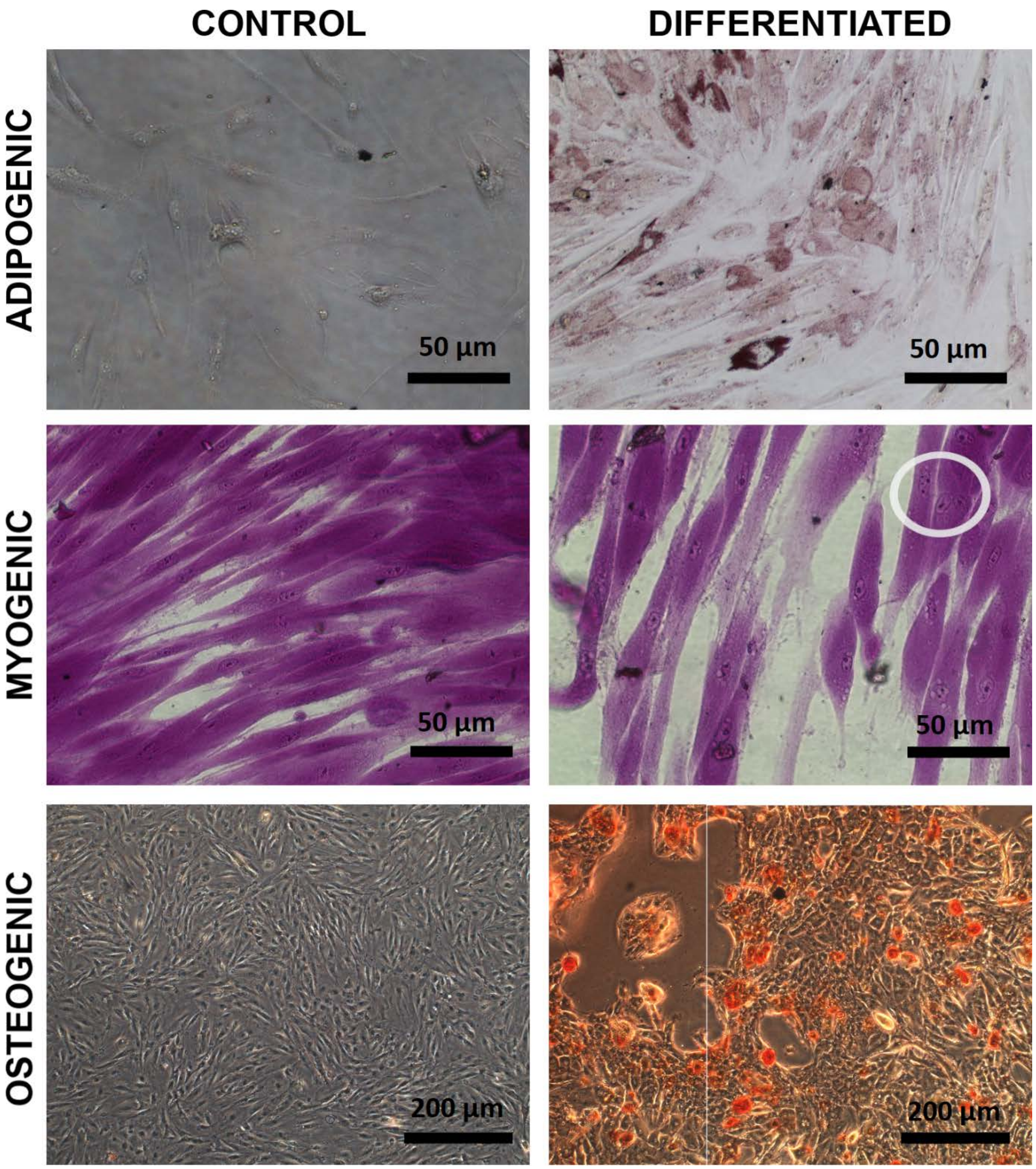

Figure S1. Evaluation MSC differentiation potential. Cells were differentiated to adipogenic, myogenic and osteogenic lineage. Control column—cells stained with the same dyes but were grown in growth media without differentiation inducing supplements, differentiated column—cells were grown in differentiation inducing media. White circle marks cells with fused/multiple nuclei after myogenic differentiation.
